# CC-223, NSC781406, and BGT226 Exerts a Cytotoxic Effect Against Pancreatic Cancer Cells via mTOR Signaling

**DOI:** 10.3389/fphar.2020.580407

**Published:** 2020-11-11

**Authors:** Yangyang Guo, Hengyue Zhu, Min Weng, Hewei Zhang, Cheng Wang, Linxiao Sun

**Affiliations:** Key Laboratory of Diagnosis and Treatment of Severe Hepato-Pancreatic Diseases of Zhejiang Province, Zhejiang Provincial Top Key Discipline in Surgery, Wenzhou Medical University First Affiliated Hospital, Wenzhou, China

**Keywords:** CC-223, NSC781406, BGT226, mTOR, pancreatic cancer

## Abstract

The mTOR signaling pathway is abnormally activated in pancreatic cancer and is related to tumor glucose metabolism. However, its specific regulation mechanism is still unclear. Therefore, this study aims to investigate whether Sestrin2 affects the glucose metabolism of pancreatic cancer by modulating mTOR signal and then affects its biological behavior. We have observed that l-leucine can promote the proliferation of pancreatic cancer cells and increase the expression of Sestrin2 and p-mTOR proteins. In order to further study the role of Sestrin2 and mTOR signaling in pancreatic cancer, we conducted Sestrin2 overexpression and mTOR pharmacological inhibition experiments. We found that Sestrin2 overexpression can increase glycolysis of pancreatic cancer cells and promote their proliferation. This effect can be eliminated by mTOR inhibitors. Finally, we found that Sestrin2 knockdown could inhibit the growth of pancreatic cancer *in vivo*. In conclusion, these findings suggest that Sestrin2 may promote the occurrence and development of pancreatic cancer through mTOR signaling.

## Introduction

Pancreatic cancer is one of the severe tumors in digestive tract with high degree of malignancy, early metastasis and poor prognosis (Tsumura et al., 2019). It is known that the majority of people are diagnosed with pancreatic cancer at an advanced stage, which elevates the chance of relapse and renders the treatment and recovery more difficult (Herman et al., 2008). Although numerous advanced treatments, including medications, surgical removal of tumors and targeted-radiation therapy, have been widely applied in patients with pancreatic cancer, the overall 5-year survival rate is still less than 5% (Kong et al., 2009). Thus, it is crucial to develop effective therapeutic targets for pancreatic cancer.

Abnormal activation of signal transduction pathways related to tumor cell metabolism plays an important role in tumorigenesis and development, such as PI3K/AKT/mTOR signaling pathway. Most cancer cells can maintain a high degree of glycolysis even under aerobic conditions, which called “Warburg effect.” This way can maintain the continuous proliferation of cancer cells. Jiao et al. reported that curcumin inhibits the proliferation of lung cancer via PI3K/mTOR signaling pathway. Therefore, targeted inhibition of this pathway is expected to become a new treatment strategy for cancers.

As a family of stress-induced proteins, Sestrins are involved in the maintenance of cellular homeostasis, as well as in tumorigenesis by regulating various downstream molecules of mTOR signaling pathway (Wang et al., 2019). Emerging studies have shown that mTOR-associated pathways are closely related to cell growth and metabolism in different human disorders, including cancers (Chen et al., 2019; Chung et al., 2019; Garcia et al., 2019). l-Leucine is an amino acid that plays an important role in the protein synthesis (Vago et al., 2020). It has been reported that leucine can also be used as a signal molecule to participate in the regulation of insulin secretion (Yang et al., 2010). The combination of leucine and Sestrin2 is essential for the activation of mTOR pathway in living organisms. However, the key role of Sestrin2 in pancreatic cancer has not been reported.

In this study, we demonstrated that the functions of Sestrin2 in the development of pancreatic cancer both *in vivo* and *in vitro*. The overexpression of Sestrin2 significantly promoted the growth and proliferation of pancreatic cancer cells. In addition, l-leucine could activate Sestrin2 to promote the growth and proliferation of tumor cells. Our research also found that mTOR acts as a downstream molecule of Sestrin2, and the suppression of mTOR signaling inhibited the proliferation of pancreatic cancer cells. The knockdown of Sestrin2 also suppressed the growth of tumor in nude mice.

## Materials and Methods

### Drugs and Antibodies

mTOR inhibitors PF-04691502 (purity of 99.64%), GSK1059615 (purity of 98.91%), Dactolisib (purity of 99.13%), PI-103 (purity of 99.86%), CC-223 (purity of 99.43%), AZD-8055 (purity of 99.19%), Salidroside (purity of 98.63%), Vistusertib (purity of 98.82%), PP121 (purity of 98.89%), Apitolisib (purity of 99.26%), Voxtalisib (purity of 99.93%), Sapanisertib (purity of 99.06%), Palomid 529 (purity of 99.47%), KU-0063794 (purity of 99.23%), Torkinib (purity of 98.76%), GNE-477 (purity of 95.81%), 3BDO (purity of 99.67%), PQR620 (purity of 98.06%), HDACs (purity of 99.12%), CZ415 (purity of 98.43%), mTOR inhibitor-1 (purity of 99.29%), Omipalisib (purity of 99.31%), NSC781406 (purity of 99.91%), JR-AB2-011 (purity of 98.09%), mTOR inhibitor-3 (purity of 98.54%), BGT226, Temsirolimus (purity of 99.56%), MHY1485 (purity of 99.86%), GNE-317 (purity of 99.26%), Torin 2 (purity of 99.93%), ETP-46464 (purity of 99.13%), WYE-354 (purity > 98.0%), Dihydromyricetin (purity of 99.54%), CC-115 (hydrochloride, purity of 96.64%), GDC-0349 (purity of 98.2%), LY3023414 (purity of 99.77%), WAY-600 (purity of 95.12%), GNE-493 (purity of 95.12%), PI-103 (hydrochloride, purity of 99.86%), XL388 (purity of 98.46%), GDC-0084 (purity of 99.28%), CC-115 (purity of 96.64%), Bimiralisib (purity of 98.90%), WYE-132 (purity of 98.98%), and VS-5584 (purity of 98.01%) were purchased from MCE (China). All drugs were dissolved in DMSO as 20 mM. mTOR antibody (ab2732) was purchased from Abcam (Beijing, China). S6K1 antibody (CST 9202), p-S6K1 antibody (CST 9204S), and p-mTOR antibody (CST 5536S) were purchased from Cell Signaling Technology (CST, USA). GAPDH antibody (AP0063) was purchased from Bioworl Technology (USA).

### Cell Lines

The human pancreatic cancer cell line PANC-1(CRL-1469), BxPC-3(CRL-1687), SW1990(CRL-2172), Patu8988(PTA-8988), and CFPAC-1(CRL-1918) were purchased from American type culture collection (ATCC, Manassas, VA, USA). PANC-1 and Patu8988 cells were cultured in DMEM containing 10% fetal bovine serum (FBS, Calabasas, CA), 100 U/ml penicillin and 100 µg/ml streptomycin. BxPC-3, SW1990, and CFPAC-1 cells were cultured in 1640 medium containing 10% FBS, 100 U/ml penicillin, and 100 µg/ml streptomycin. All cells were maintained at 37°C and 5% humidified CO_2_ incubator.

### Real Time Cell Analysis

Proliferation of PANC-1 cells was measured by RTCA. 1.5–2 × 10^4^ cells were seeded into a 16-well plate (E-plate) with 200 µL growth media and cultured for 36–48 h at 37°C. The cell growth index was automatically recorded every 15 min using Real-time Cellular Analysis System (Roche, Penzberg, Germany).

### Wound Healing Assay

The exponentially growing PANC-1 cells were seeded in 6-well plates and incubated for 24 h at 37°C. Then, the culture area was scratched using a crystal pipette tip to make a linear gap. Detached cells were washed away with PBS and medium with CC-223, NSC781406, or BGT226 (20 μM) was then added. Cells were allowed to fill the gap and images of the culture area were captured duly using microscope per 24 h.

### Transwell Invasive Assay

Transwell invasive assay was used to detect the invasion ability of PANC-1 cells *in vitro*. PANC-1 cells at a concentration of 2 × 10^5^ cells in 200 μL of serum-free medium with CC-223, NSC781406, or BGT226 (20 μM) were inoculated in the upper chamber, coated with Matrigel^®^, and medium containing 10% FBS was then added to the lower chamber as a chemoattractant. After incubation for 24 h at 37°C, the cells that invase through the filters into the lower chamber were fixed with 4% paraformaldehyde for 20–30 min and then stained with 0.5% crystal violet for 5 min at room temperature. The number of cells in six randomly selected fields (200×) from each well was counted under an optical microscope.

### Colony Formation Assay

The PANC-1 cells were plated in 12-well plates at a density of 400 cells/well. The CC-223, NSC781406, or BGT226 (20 μM) were then added into each well. Once the cells grow into colonies that are visible to the naked eye after 10–15 days, colonies were washed with PBS and fixed with formaldehyde. Staining with crystal violet was carried out to count the number of colonies.

### Lentiviral Transduction

PANC-1 cells were transduced with pLEGO-iG-U6 NFE2 shDNA or a scrambled control as previously described. Green fluorescent protein (GFP)–expressing cells were sorted to obtain the correct populations.

### Western Blotting

The expression levels of proteins were measured by using western blotting. Proteins were firstly extracted in accordance with the manufacture’s protocol, and the concentration of proteins was determined using BCA protein assay kit (Beyotime, Shanghai, China). 35  µL protein of each sample was loaded onto 10% SDS–PAGE Gel for the western blotting analysis. Membranes were incubated overnight with the appropriate primary antibodies at 4°C after blocking membranes with 5% skim milk for 2 h at 25°C, followed by 5 min washing of membranes with TBST for five times. The secondary antibodies (1:5,000) were used for 1 h of incubation at 25°C. After another five times washing with TBST, protein bands were visualized by an enhanced chemiluminescent reagent (Thermo Fisher).

### Oxidative Phosphorylation and Glycolysis Assay

The cellular oxygen consumption rate (OCR) and extracellular acidification rate (ECAR) were determined via Seahorse XF96 Extracellular Flux Analyser (Seahorse Bioscience, North Billerica, MA, USA). 2.5 × 10^4^ of PANC-1 cells were cultured in 96-well plates and incubated overnight at 37°C with 5% humidified CO_2_. After that, PANC-1 cells were pretreated with CC-223, NSC781406, and BGT226 (20 μM) for 24 h. At the same time, the calibration plates were incubated overnight at 37°C. The assay medium was used instead of the cell medium. The probe plate was replaced by the cell plate after probe calibration. The compounds were then injected sequentially as per the manufacturer’s instructions.

### Animal Experiments

The animal experiments were carried out in accordance with the guidelines of the Institutional Animal Care and Use Committee, University Laboratory Animal Research of Wenzhou Medical University. Male nude mice were put under specific pathogen-free conditions, followed by inject PANC-1 cells (Sestrin2 knockdown or not, 5 × 10^6^) subcutaneously into the left flank of the 5-week-old mice (*n* = 12). The growth of tumor was monitored every 3 days by measuring the length and width using a digital caliper. The tumor volume was then calculated according to the following formula: volume = (length × width^2^)/2. After 30 days, the mice were sacrificed and photographed, and the tumors were dissected and weighed.

### Statistical Analysis

Statistical analysis was performed with GraphPad Prism 6.0 (GraphPad Software Inc., San Diego, CA, USA). All data were presented as mean ± standard deviation. One-way ANOVA and the Student-Newman Keuls tests were used to compare means of each groups. *p* < 0.05 was considered as statistically significant. Analysis between pair groups were also performed using the LSD method once the analysis of variance were statistically significant.

## Results

### l-Leucine Promotes Growth and Proliferation of PANC-1 Cells

**FIGURE 1:**
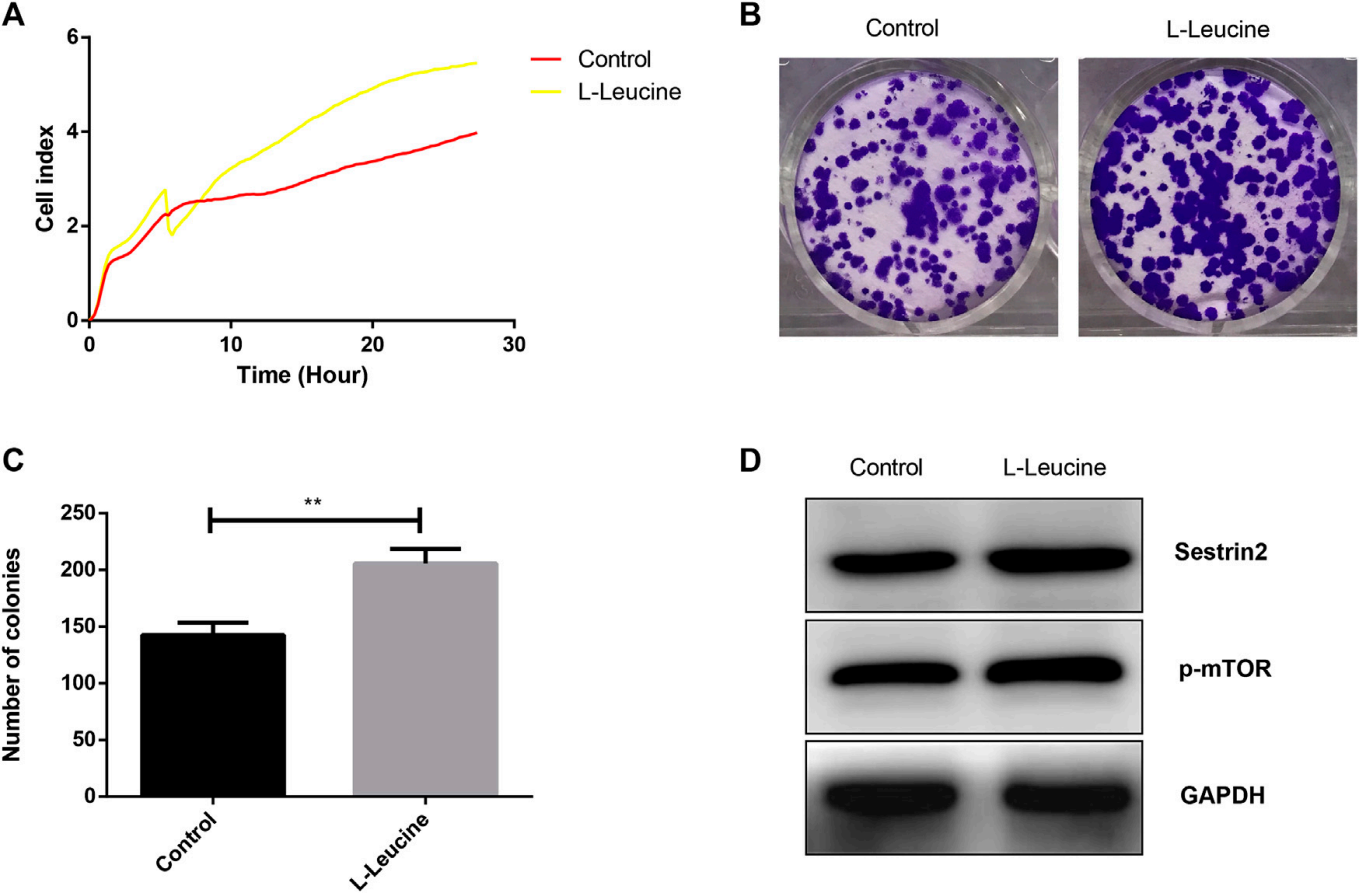
l **-**Leucine promotes growth and proliferation of PANC-1 cells. **(A)** RTCA assay. Cells were treated with 50 μM l **-**leucine and cell index were automatically recorded every 15 min. **(B)** Colony formation assay. Cells were treated with 50 μM l-leucine and incubated for 14 days. **(C)** Statistical bar chart of the number of clones. **(D)** Western blot analysis of Sestrin2 and p-mTOR. **p < 0.01.

The effects of l-leucine on the growth and proliferation of PANC-1 cells were evaluated with unlabeled RTCA and colony formation assay. As showed in Figure 1A, a significantly increased proliferation of PANC-1 cells was found after the treatment with 50 μM l-leucine. In addition, the number of colonies of PANC-1 cells with l-leucine was relatively higher than that of the control group (Figures 1B,C). Furthermore, l-leucine promoted the expression of Sestrin2 and p-mTOR proteins (Figure 1D). These findings suggested that l-leucine could promote the proliferation and clonogenicity of PANC-1 cells.

### mTOR Inhibitors Suppress Proliferation, Invasion and Migration of PANC-1 Cells via mTOR Signaling

**FIGURE 2:**
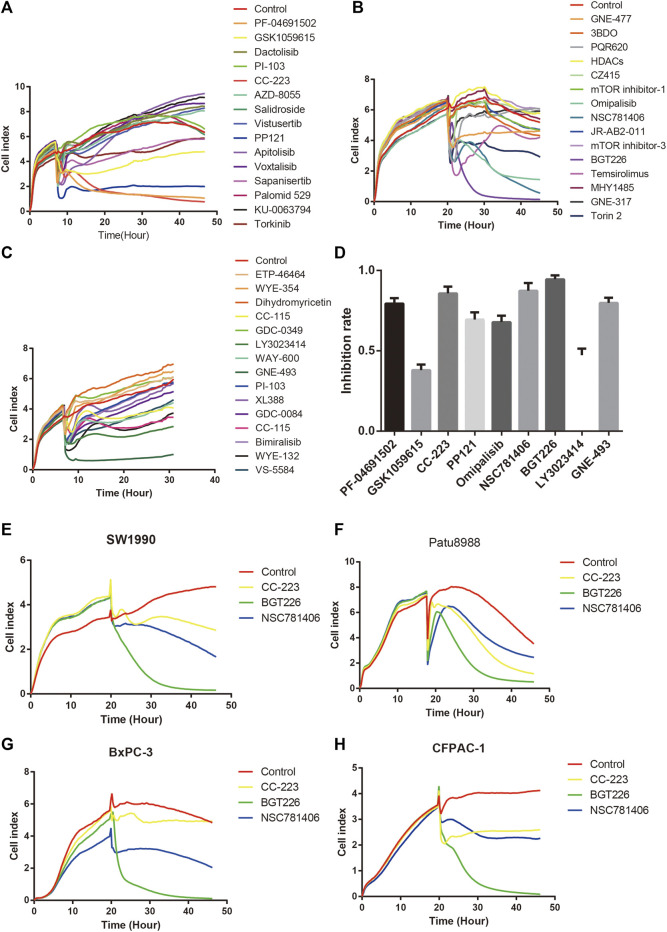
Effects of mTOR inhibitors on the proliferation of pancreatic cancer cells. **(A)** RTCA assay. Cells were treated with PF-04691502, GSK1059615, Dactolisib, PI-103, CC-223, AZD-8055, Salidroside, Vistusertib, PP121, Apitolisib, Voxtalisib, Sapanisertib, Palomid 529, KU-0063794, and Torkinib (20 μM). **(B)** RTCA assay. Cells were treated with GNE-477, 3BDO, PQR620, HDACs, CZ415, mTOR inhibitor-1, Omipalisib, NSC781406, JR-AB2-011, mTOR inhibitor-3, BGT226, Temsirolimus, MHY1485, GNE-317, and Torin 2 (20 μM). **(C)** RTCA assay. Cells were treated with ETP-46464, WYE-354, Dihydromyricetin, CC-115 (hydrochloride), GDC-0349, LY3023414, WAY-600, GNE-493, PI-103 (hydrochloride), XL388, GDC-0084, CC-115, Bimiralisib, WYE-132, and VS-5584 (20 μM). **(D)** A statistical bar chart of RTCA results of mTOR inhibitors. **(E)** RTCA assay. SW1990 cells were treated with CC-223, NSC781406, and BGT226 (20 μM). **(F)** RTCA assay. Patu8988 cells were treated with CC-223, NSC781406, and BGT226 (20 μM). **(G)** RTCA assay. BxPC-3 cells were treated with CC-223, NSC781406, and BGT226 (20 μM). **(H)** RTCA assay. CFPAC-1 cells were treated with CC-223, NSC781406, and BGT226 (20 μM).

**FIGURE 3:**
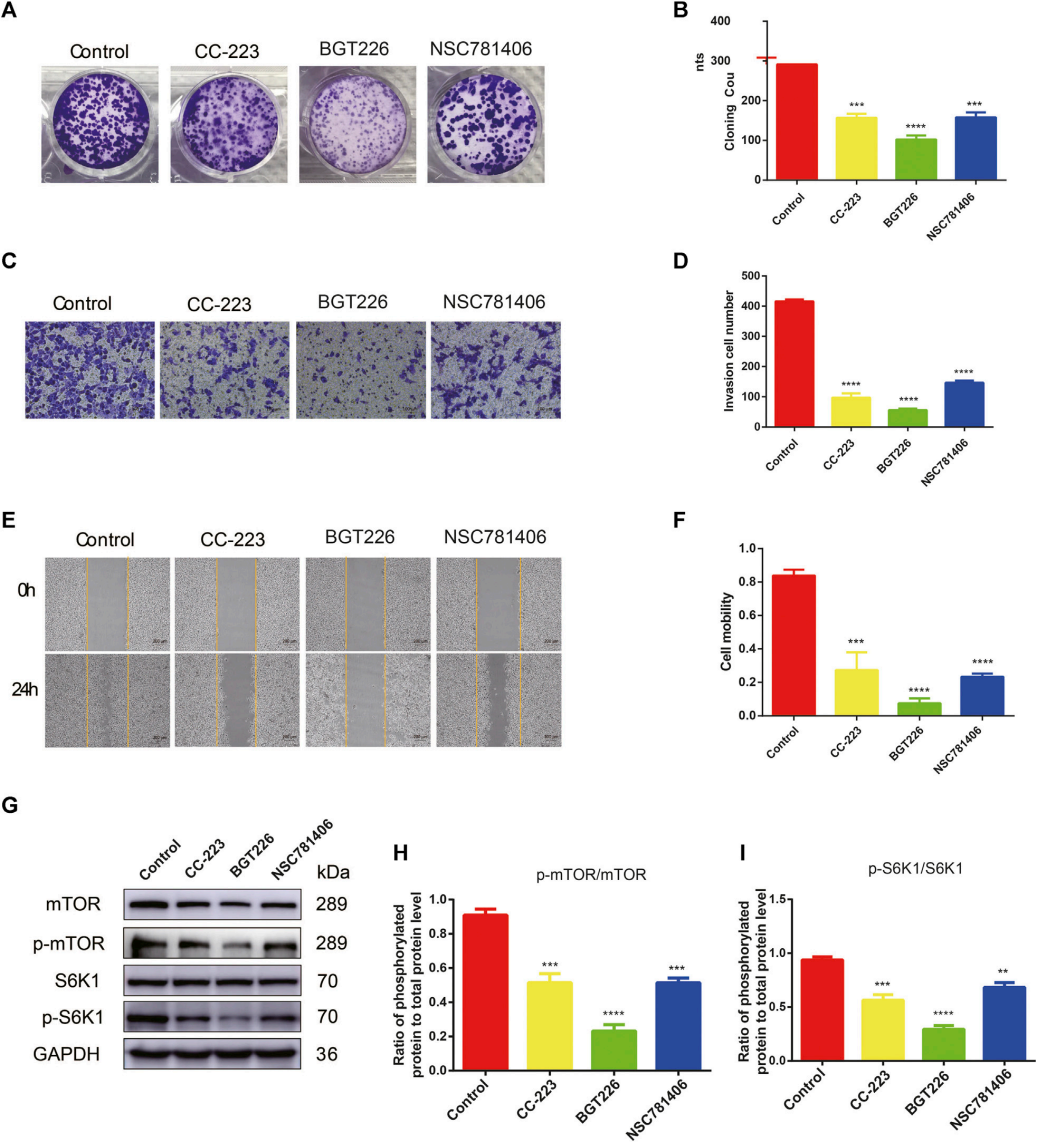
CC-223, NSC781406, and BGT226 inhibit the proliferation, invasion and migration of PANC-1 cells via mTOR/S6K1 signaling. **(A)** Colony formation assay. Cells were treated with 20 μM CC-223, NSC781406, and BGT226 and incubated for 14 days. **(B)** Statistical bar chart of the number of clones. **(C)** Transwell invasive assay. Cells were treated with 20 μM CC-223, NSC781406, and BGT226 and incubated for 24 h. **(D)** Statistical bar chart of the number of invasive cells. **(E)** Wound healing assay. Cells were treated with 20 μM CC-223, NSC781406, and BGT226 and incubated for 24 h. **(F)** Statistical bar chart of cell mobility. **(G)** Western blot analysis of proteins involved in mTOR/S6K1 signaling in PANC-1 cells. **(H)** Statistical bar chart of p-mTOR/mTOR. **(I)** Statistical bar chart of p-S6K1/S6K1. **p < 0.01; ***p < 0.001; ****p < 0.0001.

To determine the inhibitory effect of mTOR inhibitors on pancreatic cancer, RTCT test and colony formation assay was performed. PANC-1 cells were treated with 45 different types of mTOR inhibitors (20 μM) to detect the ability of cell proliferation. As demonstrated in Figures 2A–D, the proliferation ability of PANC-1 cells declined significantly after treatment with PF-04691502, GSK1059615, CC-223, PP121, Omipalisib, NSC781406, BGT226, LY3023414, and GNE-493 within 24 h, while the rest of mTOR inhibitors had no obvious inhibitory effect. Moreover, the inhibition of CC-223, NSC781406, and BGT226 were significant among all the inhibitors. Owing to exclude the specificity of cell lines, we selected four other pancreatic cell lines to verify. Consistently, the proliferation ability of SW1990, Patu8988, BxPC-3, and CFPAC-1 cells reduced within 24 h after treatment with CC-223, NSC781406, and BGT226 inhibitors (Figures 2E,H). Colony formation assay confirmed that CC-223, NSC781406, and BGT226 inhibitors constrained the proliferation and clone formation of PANC-1 cells (Figures 3A,B).

Furthermore, to investigate the effects of CC-223, NSC781406, and BGT226 (20 μM) inhibitors on the invasion of pancreatic cancer cells, transwell experiment was carried out. As shown in Figures 3C,D, compared with the control group, the invasion number of PANC-1 cells was dramatically diminished after treatment with CC-223, NSC781406, and BGT226, especially BGT226 inhibitor that had the strongest inhibitory effect. The migration capacity of the PANC-1 cells was attenuated after treated with above three inhibitors. Furthermore, wound healing assay results revealed that CC-223, NSC781406, and BGT226 inhibitors remarkably impeded the migration of PANC-1 cells (Figures 3E,F). In particular, the inhibitory effect of BGT226 was noticeable. These results collectively indicated that CC-223, NSC781406, and BGT226 could restrain the invasion and migration abilities of PANC-1 cells.

The expression of mTOR signaling channel proteins was detected using western blotting to investigate the potential mechanisms of CC-223, NSC781406, and BGT226 inhibitors on pancreatic cancer. From Figures 3G–I, the application of CC-223, NSC781406, and BGT226 (20 μM) inhibitors downregulated the expression of mTOR, p-mTOR, and p-S6K1, while S6K1 had no significant change, suggesting that mTOR/S6K1 signaling was involved in the CC-223, NSC781406, and BGT226 inhibition.

### CC-223, NSC781406, and BGT226 Inhibit Glycolysis in Pancreatic Cancer Cells

**FIGURE 4:**
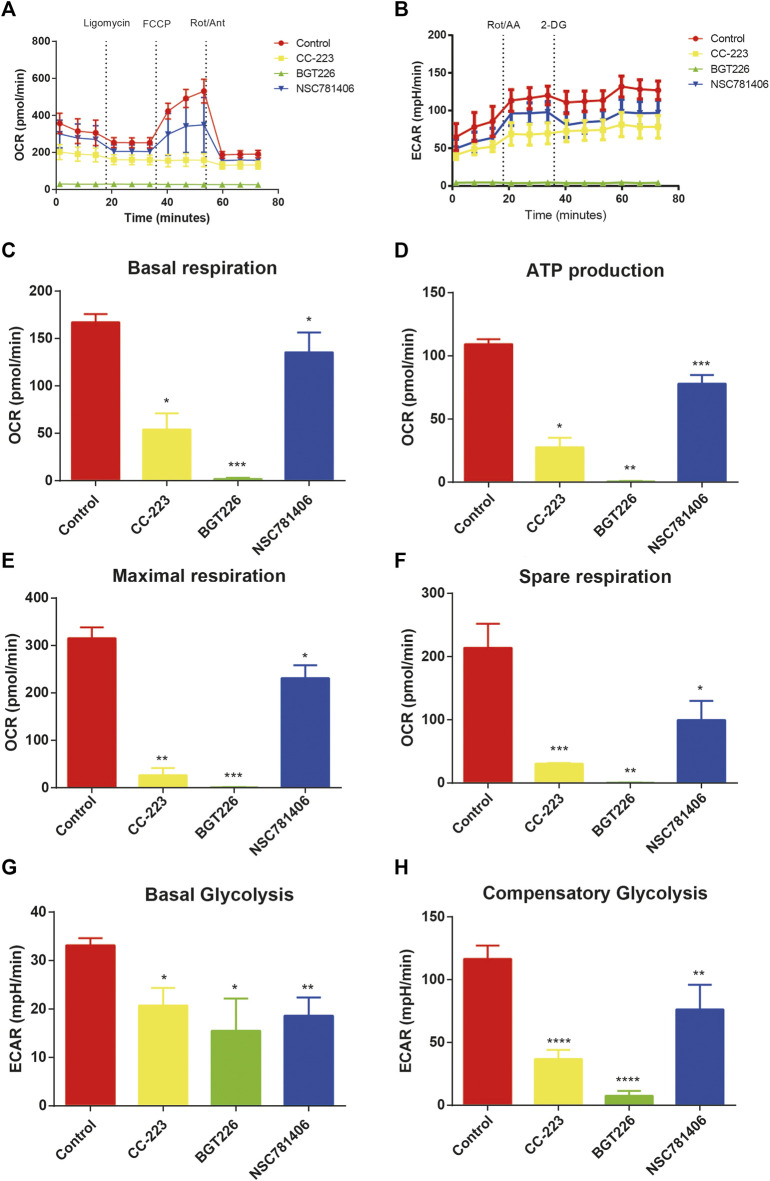
CC-223, NSC781406, and BGT226 inhibit glycolysis in PANC-1 cells. **(A)** OCR assay. Cells were treated with 20 μM CC-223, NSC781406, and BGT226 for 24 h. **(B)** ECAR assay. Cells were treated with 20 μM CC-223, NSC781406, and BGT226 for 24 h. **(C)** Statistical bar chart of the basal respiration. **(D)** Statistical bar chart of the ATP production. **(E)** Statistical bar chart of the maximal respiration. **(F)** Statistical bar chart of the spare respiration. **(G)** Statistical bar chart of the basal glycolysis. **(H)** Statistical bar chart of the compensatory glycolysis. *p < 0.05; **p < 0.01; ***p < 0.001; ****p < 0.0001.

To explore the efficacy of CC-223, NSC781406, and BGT226 inhibitors on cellular bioenergetics of PANC-1, we analyzed ECAR and OCR in real time of PANC-1 cells treated with CC-223, NSC781406, and BGT226. The result indicated that the treatment of mTOR inhibitors resulted in the significant reduction of ECAR and OCR in PANC-1 cells (Figures 4A,B). In addition, we analyzed different parameters of erobic glycolytic rate, and found that these three mTOR inhibitors led to decreased basal respiration, ATP production, maximal respiration, spare respiration, basal glycolysis, and compensatory glycolysis in PANC-1 cells (Figures 4C–H). These data clarified that CC-223, NSC781406, and BGT226 could block glycolysis in pancreatic cancer cells.

### Sestrin2 Promotes Pancreatic Cancer Cell Growth and Proliferation *In Vitro*

**FIGURE 5:**
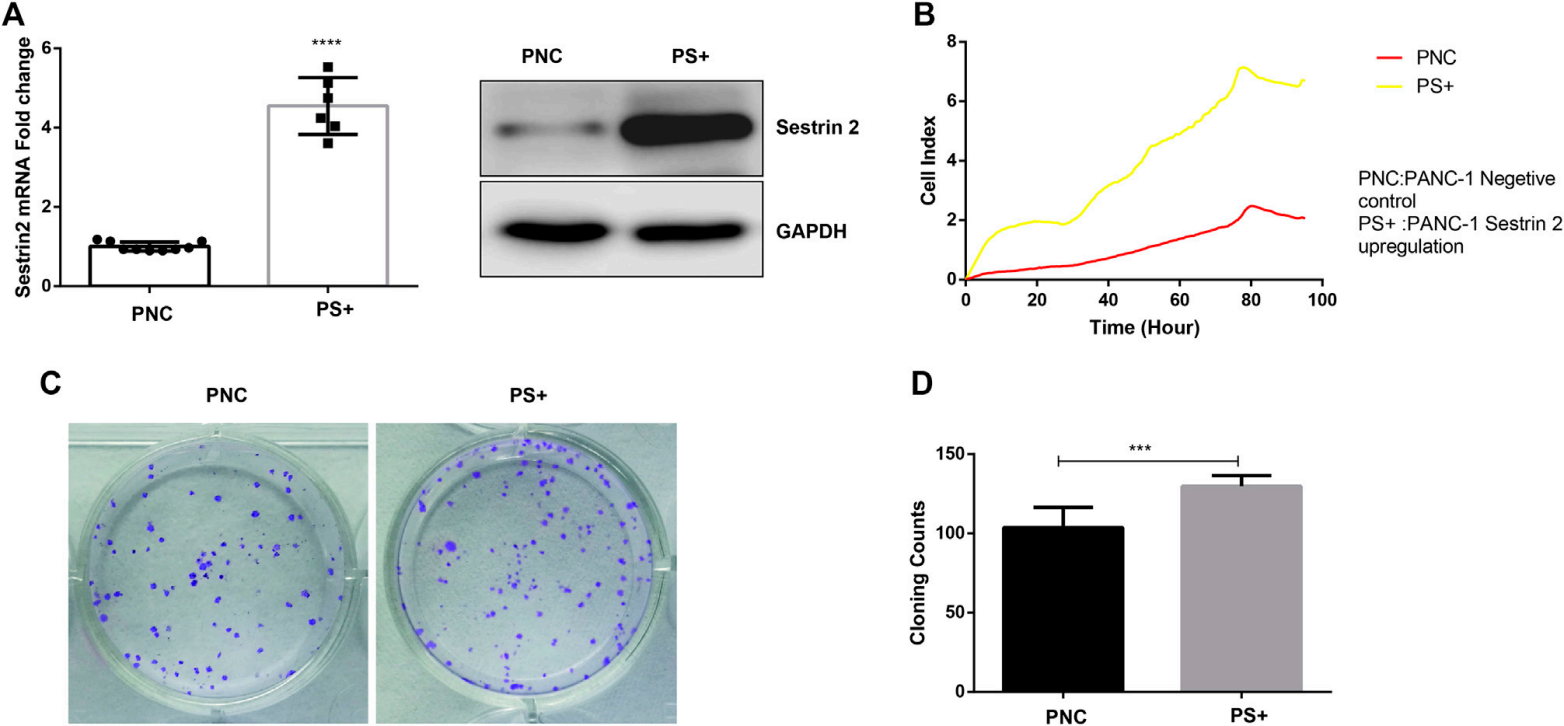
Sestrin2 overexpression promotes the growth and proliferation of PANC-1 cells. **(A)** qRT-PCR and WB analysis of Sestrin2 in PANC-1 cells after Sestrin2 overexpression. **(B)** RTCA assay. Sestrin2 overexpression or not of PANC-1 cells were incubated for about 96 h and cell index were automatically recorded every 15 min. **(C)** Colony formation assay. Sestrin2 overexpression or not of PANC-1 cells were incubated for 14 days. **(D)** Statistical bar chart of the number of clones. ***p < 0.001.

To assess the role of Sestrin2 in pancreatic cancer cells, we constructed an overexpression model of Sestrin2 to detect growth and proliferation of PANC-1 cells through RTCA and colony formation test. qRT-PCR and western blot revealed the upregulation of protein and mRNA levels of Sestrin2 (Figure 5A). As showed in Figure 5B, Sestrin2 overexpression noticeably facilitated the proliferation of PANC-1 cells compared to the control group. Consistent with the result from RTCA, plate colony formation test also showed that the number of colonies in the overexpression group remarkably higher than that of in the control group (Figures 5C,D), suggesting that Sestrin2 could promote the proliferation and clonogenicity of PANC-1 cells.

### Overexpression of Sestrin2 Promotes Glycolysis and Mitochondrial Respiration of PANC-1 Cells

**FIGURE 6:**
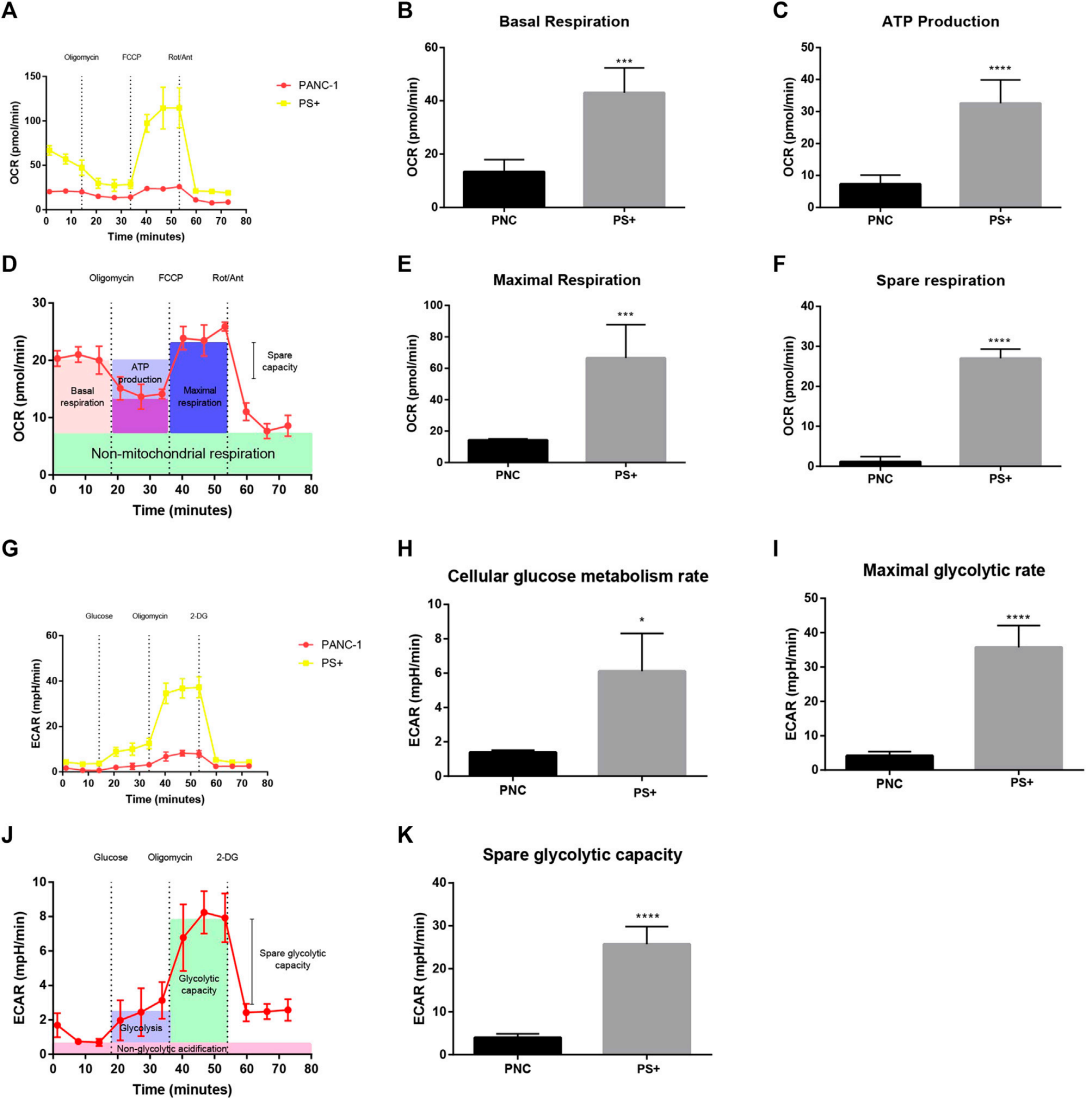
Sestrin2 overexpression promotes glycolysis in PANC-1 cells. **(A)** OCR assay. Sestrin2 overexpression or not of PANC-1 cells were incubated for 24 h. **(B)** Statistical bar chart of the basal respiration. **(C)** Statistical bar chart of the ATP production. **(D)** Schema chart of OCR. **(E)** Statistical bar chart of the maximal respiration. **(F)** Statistical bar chart of the spare respiration. **(G)** ECAR assay. Sestrin2 overexpression or not of PANC-1 cells were incubated for 24 h. **(H)** Statistical bar chart of the cellular glucose metabolism rate. **(I)** Statistical bar chart of the maximal glycolytic rate. **(J)** Schema chart of ECAR. **(K)** Statistical bar chart of the spare glycolytic capacity. *p < 0.05; ***p < 0.001; ****p < 0.0001.

To further explore the effect of Sestrin2 on the cellular bioenergetics of pancreatic cancer cells, extracellular flux analyzer was adopted to estimate the ECAR and OCR of PANC-1 cells with or without Sestrin2 overexpression. As shown in Figures 6A,D, the overexpression of Sestrin2 resulted in enhanced cellular OCR and ECAR in PANC-1 cells. The various parameters of OCR and ECAR revealed that Sestrin2 overexpression led to the increase of basal respiration (Figure 6B), ATP production (Figure 6C), maximal respiration (Figure 6E), spare respiration (Figure 6F), cellular glucose metabolism rate (Figure 6H), maximal glycolytic rate (Figure 6I), and spare glycolytic capacity (Figure 6K). These data indicated that Sestrin2 overexpression promoted the mitochondrial respiration and glycolysis in pancreatic cancer cells.

### mTOR Inhibitors Reverses the Effect of Sestrin2 Overexpression on the Biological Behavior of Pancreatic Cancer

**FIGURE 7:**
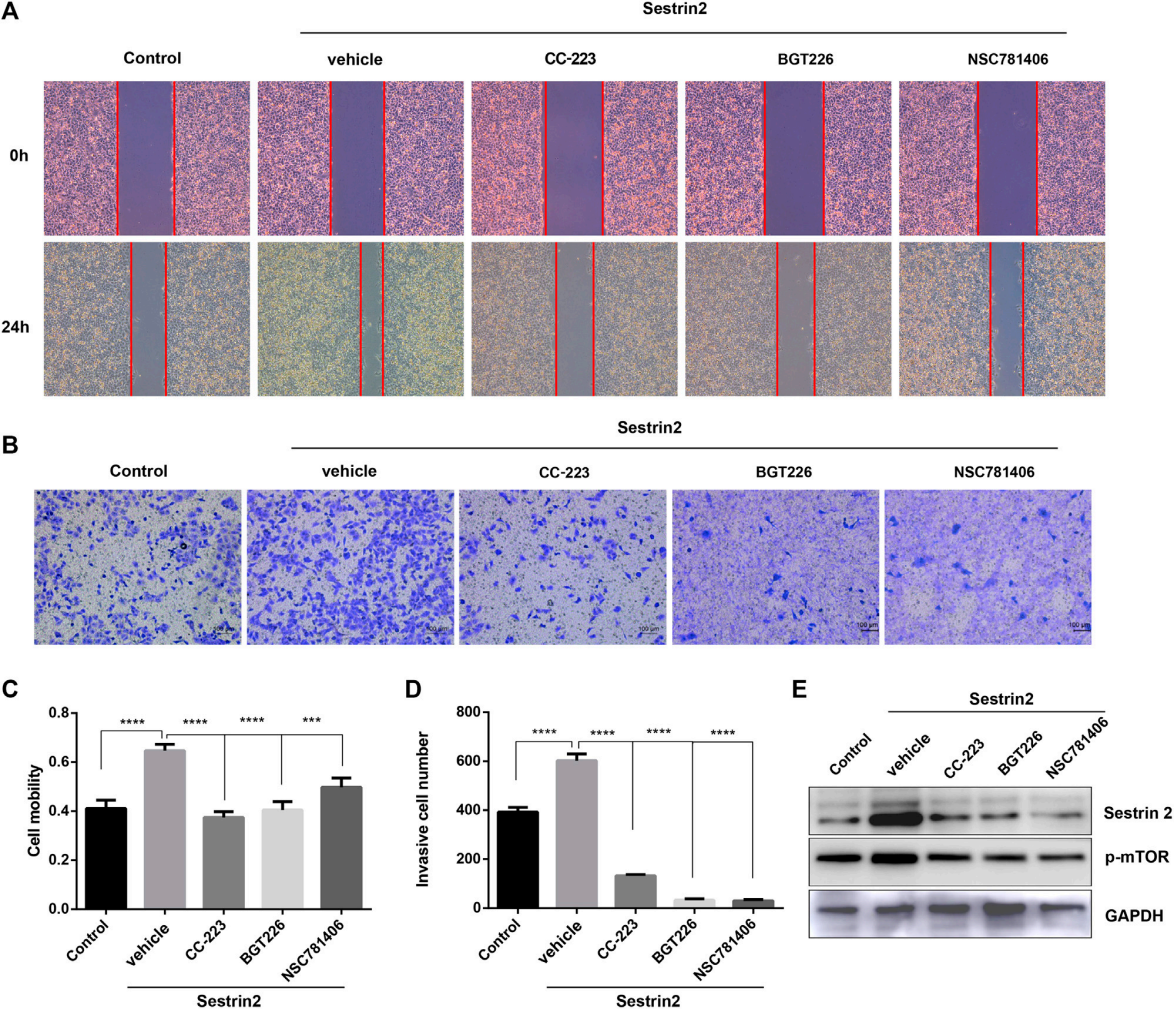
mTOR inhibitors reversed the effect of Sestrin2 overexpression on the biological behavior of pancreatic cancer. **(A,C)** Wound healing assay. PANC-1 cells with Sestrin2 overexpression or not were treated with 20 μM CC-223, NSC781406, and BGT226 and incubated for 24 h. **(B,D)** Transwell assay. PANC-1 cells with Sestrin2 overexpression or not were treated with 20 μM CC-223, NSC781406, and BGT226 and incubated for 24 h. **(E)** Western blot analysis of Sestrin2 and p-mTOR. ***p < 0.001; ****p < 0.0001.

Next, we tested the effect of mTOR inhibitors on the biological behavior of PANC-1 cells with Sestrin2 overexpression. As shown in Figures 7A,C, Sestrin2 overexpression promoted the migration ability of PANC-1 cells, which could be reversed by mTOR inhibitors. Similarly, mTOR inhibitors reversed the promotion of Sestrin2 overexpression on the invasion ability of PANC-1 cells (Figures 7B,D). Western blot shown that Sestrin2 overexpression increased the protein level of p-mTOR, while mTOR inhibitors decreased the expression of Sestrin2 and p-mTOR (Figure 7E). These data revealed mTOR inhibitors reversed the effect of Sestrin2 overexpression on the biological behavior of pancreatic cancer.

### Sestrin2 Knockdown Restrains Pancreatic Cancer Cell Growth and Proliferation *In Vivo*

**FIGURE 8:**
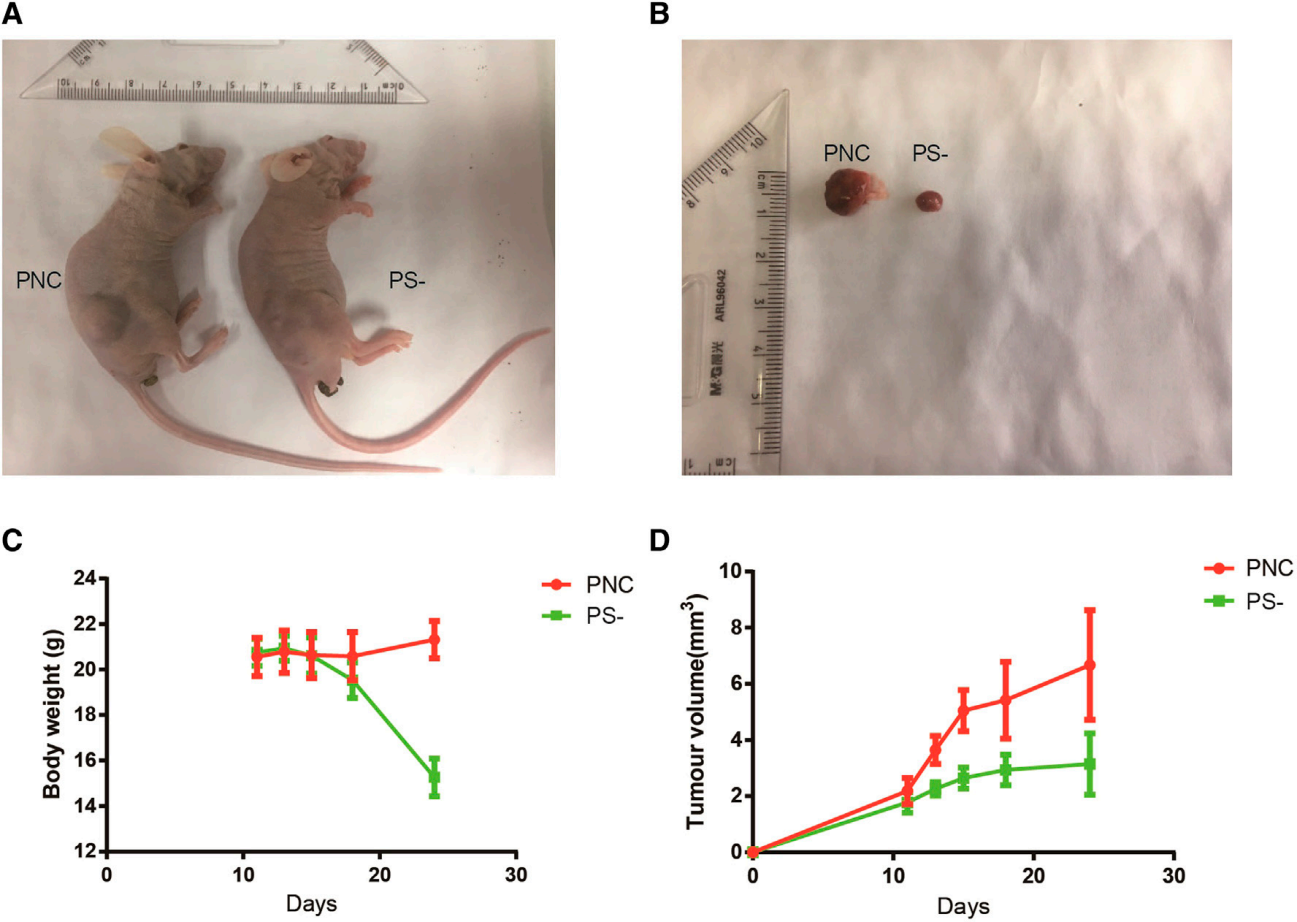
Knockdown of Sestrin2 inhibits the growth of pancreatic cancer *in vivo*. **(A)** Picture of nude mice in control group and Sestrin2 knockdown group. **(B)** Picture of tumor in control group and Sestrin2 knockdown group. **(C)** Body weight of nude mice in control group and Sestrin2 knockdown group. **(D)** Tumor volume in control group and Sestrin2 knockdown group.

To evaluate the effects of Sestrin2 on pancreatic cancer cell growth *in vivo*, PANC-1 cells with Sestrin2 knockdown were implanted subcutaneously into the nude mice. From Figures 8A,B, the knockdown of Sestrin2 markedly inhibited tumor growth *in vivo* compared to the control group. The Sestrin2 knockdown group also exhibited significant decrease of tumor volume and weight (Figures 8C,D). Overall, these results were consistent with *in vitro* findings, indicating that Sestrin2 could promote the growth of pancreatic cancer cells *in vivo*.

## Discussion

Pancreatic cancer is one of the fatal malignancy characterized by high aggressive and high drug resistance (Nie et al., 2020). The therapeutic effects of surgery, radiotherapy, and chemotherapy are not satisfied due to most patients are diagnosed as advanced (Baguña Torres et al., 2020). Despite various studies on pancreatic cancer, the underlying mechanism of pancreatic cancer development remains insufficient. Therefore, it is necessary to study the pathogenesis of pancreatic cancer and discover new therapeutic targets.

Sestrins are induced by a variety of stresses such as DNA damage, hypoxia, and metabolic changes (Dai et al., 2018). Previous studies have shown that most forms of cancer are associated with the down-regulation of Sestrin2 (Chen et al., 2016; Seo et al., 2016). Induction of Sestrin2 in different cancer cell lines has shown the inhibition of oxidative stress and slow tumor formation (Budanov, 2011; Ding et al., 2015). However, the role of Sestrin2 in pancreatic cancer is not well-defined. In this study, we found that the overexpression of Sestrin2 can significantly promote the proliferation of PANC-1 cells *in vitro*, as well as the glycolysis and mitochondrial respiration. Nevertheless, the knockdown of Sestrin2 inhibited the growth and proliferation of pancreatic cancer cells. These results suggested that the Sestrin2 gene played an important role in inhibiting the growth of pancreatic cancer cells.

mTOR consists of two distinct multi-protein complexes including rapamycin complex mechanism target 1 (mTORC1), which is sensitive to rapamycin, and mTORC2, which is insensitive to rapamycin (Nacarelli et al., 2018; Royce et al., 2019). mTORC1 protein kinase is considered as a major regulator because it responds to various stimuli, including growth factors, oxidative stress, and changes in energy levels (Chang et al., 2014; Souder and Anderson, 2019). mTORC1 stimulation can cause phosphorylation of p70 ribosomal protein S6 kinase (P70S6K) and 4E binding protein-1 (4EBP1), suggesting increased protein synthesis, lipid synthesis, cell proliferation, and cell survival (Aboudehen et al., 2018). Sustained mTOR stimulation is associated with various diseases, such as diabetes, obesity, cardiovascular disease, cancer, and autoimmune diseases (Chang et al., 2014; Cunningham et al., 2018). We confirmed through multiple pancreatic cell lines that mTOR inhibitors restrained the proliferation, invasion, and migration of PANC-1 cells by downregulating the expression of mTOR, p-mTOR, and p-S6K1, which was consistent with the previous studies. Additionally, the metabolism of tumor cells is different from normal cells due to glycolysis (Peng et al., 2014). Three effective mTORC inhibitors (CC-223, NSC781406, and BGT226) exhibited significant suppression of glycolysis in pancreatic cancer cells, reducing the energy source and constrained proliferation, invasion and migration of pancreatic cancer cells. Furthermore, Sestrins have been considered as a negative regulator of mTORC1 pathway (Boutouja et al., 2019). Several reports have pointed out that Sestrins block mTORC1 pathway through the activation of tuberous sclerosis (TSC) and AMP-activated protein kinase (AMPK) (Boutouja et al., 2019). Sestrins is a member of the upstream growth factor sensing branch of mTOR. In recent years, it is known that Sestrin2 is the leucine sensor of mTORC1 pathway, while mTORC1 upstream of Sestrins negatively regulates GATOR2, which is a positive regulator of the nutrient sensing branch (Budanov and Karin, 2008; Menon et al., 2014). However, the exact molecular function of GATOR remains to be explored. We speculate that under the deprivation of leucine, Sestrin2 binds to GATOR2 may potentially suppress mTORC1 pathway. Meantime, leucine stimulation leads to the dissociation of Sestrin2 from GATOR2, which may alleviate its inhibition toward mTOR (Chantranupong et al., 2014). l-Leucine promoted the proliferation and clone formation of PANC-1 cells in our study, and the mechanism may be that through this pathway, the inhibition of mTOR reduced, thus promoting the growth of pancreatic cancer cells.

## Conclusion

In summary, our results suggests that mTORC1 and nutrition-sensing molecule Sestrins may be new pharmacological targets for the treatment of pancreatic cancer, providing reference value for future researchers. Some limitations exist, and further studies are needed to clarify the underlying mechanism in greater detail.

## Data Availability Statement

The raw data supporting the conclusions of this article will be made available by the authors, without undue reservation.

## Ethics Statement

The animal study was reviewed and approved by Institutional Animal Care and Use Committee of Wenzhou Medical University, China.

## Author Contributions

All authors reviewed the manuscript. All authors have read and approved the manuscript. CW and LS designed the experiment and was responsible for writing the manuscript. YG conducted the most experiment and analyzed the results. HYZ, MW, and HWZ participated in the experiment and helped to analyze the data.

## Conflict of Interest

The authors declare that the research was conducted in the absence of any commercial or financial relationships that could be construed as a potential conflict of interest.
